# Common features and interesting differences in transcriptional responses to secretion stress in the fungi *Trichoderma reesei *and *Saccharomyces cerevisiae*

**DOI:** 10.1186/1471-2164-7-32

**Published:** 2006-02-22

**Authors:** Mikko Arvas, Tiina Pakula, Karin Lanthaler, Markku Saloheimo, Mari Valkonen, Tapani Suortti, Geoff Robson, Merja Penttilä

**Affiliations:** 1 VTT Biotechnology, Tietotie 2, Espoo, PL 1500, 02044 VTT, Finland; 2 School of Biological Sciences, University of Manchester, 1800 Stopford Building, Oxford Road, Manchester M13 9 PT, UK

## Abstract

**Background:**

Secretion stress is caused by compromised folding, modification or transport of proteins in the secretory pathway. In fungi, induction of genes in response to secretion stress is mediated mainly by the unfolded protein response (UPR) pathway. This study aims at uncovering transcriptional responses occurring in the filamentous fungi *Trichoderma reesei *exposed to secretion stress and comparing these to those found in the yeast *Saccharomyces cerevisiae*.

**Results:**

Chemostat cultures of *T. reesei *expressing human tissue plasminogen activator (tPA) and batch bioreactor cultures treated with dithiothreitol (DTT) to prevent correct protein folding were analysed with cDNA subtraction and cDNA-amplified fragment length polymorphism (AFLP) experiments. ESTs corresponding to 457 unique genes putatively induced under secretion stress were isolated and the expression pattern of 60 genes was confirmed by Northern analysis. Expression of these genes was also studied in a strain over-expressing inositol-requiring enzyme 1 (IREI) protein, a sensor for the UPR pathway. To compare the data with that of *S. cerevisiae*, published transcriptome profiling data on various stress responses in *S. cerevisiae *was reanalysed. The genes up-regulated in response to secretion stress included a large number of secretion related genes in both organisms. In addition, analysis of *T. reesei *revealed up regulation of the *cpc1 *transcription factor gene and nucleosomal genes. The induction of the cpcA and histone gene H4 were shown to be induced also in cultures of *Aspergillus nidulans *treated with DTT.

**Conclusion:**

Analysis of the genes induced under secretion stress has revealed novel features in the stress response in *T. reesei *and in filamentous fungi. We have demonstrated that in addition to the previously rather well characterised induction of genes for many ER proteins or secretion related proteins also other types of responses exist.

## Background

In eukaryotic cells after translocation to the endoplasmic reticulum (ER), the folding of secreted proteins is supported and controlled by chaperones, glycosylation enzymes and oxidoreductases. The correctly folded proteins are transported to the Golgi compartment where further modification of the proteins takes place and the proteins are thereafter secreted out of the cell. Accumulation of unfolded, misfolded or otherwise inefficiently secreted proteins or other impairing of secretion can cause stress to cells, i.e. secretion stress. Secretion stress can be induced by heterologous proteins, leading to reduced yields of proteins or by exposure of cells to various chemicals that inhibit protein folding or transport and induce strong, clearly measurable responses.

Eukaryotic cells respond in various ways to secretion stress. The best known response is the unfolded protein response (UPR) which is thought to modify and enhance the activity of the secretion pathway. In fungi, it is defined mainly through its transcriptional effects that are controlled by the sensor Ire1p and the downstream transcription factor Hac1p, as first described in *S. cerevisiae*. Ire1p splices *HAC1 *mRNA and only then Hac1p is actively translated and capable of activating its downstream genes [1]. The induction of almost 400 genes has been shown to depend on the *IRE1 *and *HAC1 *pathway [2]. Recently it has been shown that the transcription factor Gcn4p is also required for induction of majority of these genes [3].

The response to secretion stress in *T. reesei *has previously been shown to share several features in common with *S. cerevisiae*. Components of the UPR pathway have been isolated from *T. reesei *including the counterparts of the genes *IRE1 *and *HAC1*, as well as UPR target genes such as *PDI1*. In *T. reesei *and *Aspergillus niger *splicing of *hac1/hacA *mRNA and HAC1/HACA promoter binding activity has been shown [4-6]. *T. reesei *and *A. niger *exhibit also transcriptional down regulation of genes encoding secreted proteins (REpression under Secretion Stress, RESS) [7,8] which has not been described in *S. cerevisiae*, but which is likely to be functional in *Arabidopsis thaliana *[9]. Whether this response is directly dependent on UPR is currently not known.

In mammalian cells the UPR genes are regulated mainly by the actions of IRE1α and ATF6 which activate the XBP1 transcription factor that induces the UPR genes [10]. In addition, a PKR-like ER kinase (PERK) is activated by unfolded proteins in the ER and it phosphorylates the α subunit of the translation initiation factor 2 (eIF2α) [11]. Phosphorylation of eIF2α leads to attenuation of general translation initiation, but also to a concomitant translational activation of selected proteins, including ATF4 [12], a homologue of *S. cerevisiae *Gcn4p. ATF4 is similarly activated by GCN2 during amino acid deprivation. However the ATF4 mediated responses to secretion stress and amino acid deprivation appear to be distinct [12-14]. In secretion stress ATF4 transcriptionally up-regulates genes in amino acid biosynthesis related functions apparently to relieve oxidative stress caused by protein secretion stress [13].

*T. reesei *secretes large amounts of extracellular enzymes such as cellulase, which may set special demands for the capacity of its cells to fold and transport proteins. In agreement with this, the induction of cellulase production in *T. reesei *has been shown to coincide with UPR induction [15,16]. In this study we have set out to characterise further the transcriptional responses of *T. reesei *during secretion stress. Due to the lack of genome data when the work was initiated, we used cDNA-subtraction and cDNA-AFLP methods in the analysis. Responses to production of a heterologous protein and to treatment with the chemical agent DTT were studied. Expression of the genes was analyzed also in a transformant over-expressing the *ire1 *gene and displaying constitutive UPR induction. The genome sequence of *S. cerevisiae *has for long been available to the research community and the pathways for protein transport and UPR signalling are well characterised. We combined transcriptome data from publications where various treatments causing secretion stress in *S. cerevisiae *had been studied and reanalysed it to define groups of genes upregulated or downregulated, either in UPR dependent or independent manner. We provide, for the first time, such complete lists of gene groups for the research community. We compared these gene groups to the secretion stress induced genes detected by us in *T. reesei*. We discovered up regulation of the *cpc1*/gnc4/ATF4 homologue of *T. reesei *in accordance with studies made with mammals and *S. cerevisiae*. Interestingly also two nucleosome genes are up-regulated uniquely in *T. reesei *without a clear connection to cell cycle. The induction of the cpcA and histone gene H4 were shown to be induced also in cultures of *A. nidulans *treated with DTT.

## Results

### Computational reanalysis of *S. cerevisiae *transcriptome data from secretion stress causing conditions

**Figure 1:**
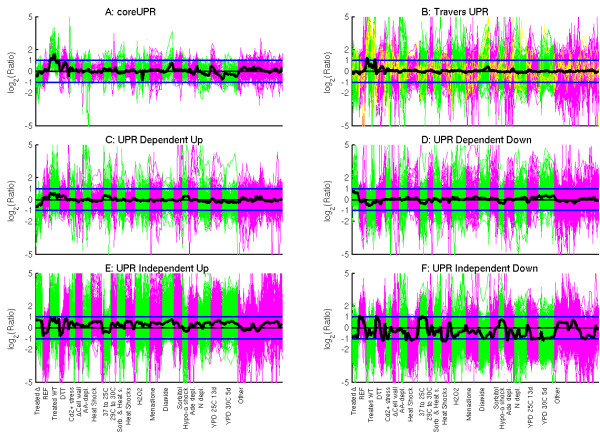
**Expression of secretion stress related genes of S. cerevisiae under different stress conditions**. Expression values of the genes (shown as log_2 _of the expression level ratio in stress vs. reference conditions) from 174 different stress conditions were collected from published articles. The expression level values of individual genes are shown in **purple**or **green**and the median of the gene group in **black**and the standard deviation of the whole experiment in **blue**. **A: **"coreUPR", genes showing significant up regulation in "**Treated WT**" experiment set against both the "**Treated ρ**" and "**REF" **experiment sets. **B: **"Travers UPR", genes defined as UPR genes and genes defined as UPR model genes in **yellow**by [2]. **C: **"UPR Dependent Up", genes showing significant up regulation only in "**Treated WT**" set against "**Treated ρ**" set. **D: **"UPR Dependent Down", genes showing significant down regulation only in "**Treated WT**" against "**Treated ρ**" set. **E: **"UPR Independent Up", genes showing significant up regulation only in "**Treated WT**" against "**REF**" set. **F: **"UPR Independent Down", genes showing significant down regulation only in "**Treated WT**" against "**REF**" set. The stress conditions were divided in sets as follows: **Treated ρ **IRE1 and HAC1 deletion strains treated with DTT or tunicamycin, used as UPR dependent reference set in this study [2, 3]; **REF **reference set, used as UPR independent reference in this study [17, 18]; **Treated WT **cultures treated with DTT or tunicamycin or recombinant strains producing a secreted heterologous protein, used as UPR conditions in this study [2, 3, 17-19, 69]; **DTT **DTT treatment, 4 h time series [17]; **Cd2+ stress **Cd^2+ ^treatment, 2 h time series and met4 deletion [48]; **ρCell Wall **strains deleted in cell wall genes [49]; **AA-depl**. methyl methanesulfonate, 3-aminotriazole and amino acid depletion treatments and GCN4 constitutive and GCN4 deletion strains [35]; **Heat Shock **1 h time series after heat shock from 25°C to 37°C, [17]; **37 to 25 **1, 5 h time series after temperature shift from 37°C to 25°C, [17]; **29C to 33C **1, 5 h time series after mild heat shock of 29°C to 33°C [17]; **Sorbi. & Heat S**. mild heat shock with different osmolarities, like previous but with sorbitol treatments [17]; **Heat Shocks **different heat shocks [17]; **H202 **2 h time series after hydrogen peroxide treatment [17]; **Menadione **2 h time series after menadione treatment [17]; **Diamide **1, 5 h time series after diamide treatment [17]; **Sorbitol **2 h time series after hyper-osmotic shock with sorbitol [17]; **Hypo-o shock **1 h time series after hypo-osmotic shock with sorbitol [17]; **Ade. depl**. 6 h time series after amino acid and adenine depletion [17]; **N depl**. 3 d time series after nitrogen depletion [17]; **YPD 25C 13 d **13 d time series of growth on YPD at 25°C [17]; **YPD 30C 5 d **5 d time series of growth on YPD at 30°C [17]; **Other **other conditions [17].

To better understand our transcriptome data on the responses to secretion stress in *T. reesei*, it was essential to evaluate the specificity of the responses to the type of stress imposed and to identify responses specific to *T. reesei *in particular. To enable reliable comparison we first combined the published data on the transcriptional responses to secretion stress related conditions in *S. cerevisiae *and analysed the data further. These were from *S. cerevisiae *cultures treated with DTT or tunicamycin to inhibit protein folding and transport [2,3,17-19], and cultures of *S. cerevisiae *producing a secreted mouse histocompatibility protein [19] (selected for the experiment set "Treated WT", Fig. 1). In order to analyse whether the observed transcriptional responses were dependent on the UPR pathway genes HAC1 and IRE1, the data sets were compared to the data from DTT and tunicamycin treatment of cultures of IRE1 and HAC1 deletion strains [2,3] (experiment set "Treated Δ", Fig. 1). In this analysis we used as reference data not yet induced early time points of DTT and heat shock time series experiments from [17] as well as two randomly picked reference cultures from [18] (experiment set "REF", Fig. 1). Genes showing significant differences in the expression between the secretion stress and reference cultures were selected using ranksum test, and the genes were divided into groups based on their expression pattern. Briefly, for each gene we tested if there was a significant difference between expression values in experiment sets "Treated WT" and "REF" and/or a significant difference between expression values in experiment sets "Treated Δ" and "Treated WT". The genes whose expression was up-regulated significantly by secretion stress (difference between "Treated WT" and "REF") and for which this change was significantly dependent of IRE1 and HAC1 (difference between "Treated Δ" and "Treated WT") were assigned to "coreUPR" (cUPR). Those genes which were only significantly dependent of IRE1 and HAC1 (difference between "Treated Δ" and "Treated WT", but no significant difference "Treated WT" and "REF"), thus showing less change, were divided by comparing the median gene expression values of experiment sets into "UPR Dependent Up" and "UPR Dependent Down". Respectively, genes which only changed significantly by secretion stress (difference between "Treated WT" and "REF"), were divided into "UPR Independent Up" and "UPR Independent Down". A detailed list of the genes in the groups is included as supplemental data. The expression of the genes in the different groups was also compared in other type of stress conditions in *S. cerevisiae*, including published data from 174 conditions. The data is summarised in Fig. 1. For comparison also the expression of the genes in the set described to be up regulated under UPR conditions IRE1 and HAC1 dependently by [2] is included.

The group of genes designated as "coreUPR" contained 46 genes up regulated significantly and UPR dependently under the secretion stress (Fig. 1A). The behaviour of this group is exceptional among the six gene groups shown in Fig. 1. It shows the least responses in the other stress conditions as the expression values of individual genes are mostly contained within the 2-fold limit (-1, 1 on the log_2 _scale plot). Interestingly it responds slightly to diamide treatment in data of [17], like the UPR gene set described by [2].

In addition to the "coreUPR" genes, a group of 135 genes showed significant dependence on UPR and were up regulated ("UPR Dependent UP", Fig. 1C). However they are not as strongly UPR-regulated, as they have no significant difference between gene expression values of experiment sets "Treated WT" and "REF". 155 genes showed UPR dependence and were down regulated ("UPR Dependent Down", Fig. 1D). Some of the genes in the groups "UPR Dependent Up" and "UPR Dependent Down" genes showed responses also in other stress conditions. In many cases the responses of the up regulated and down regulated genes were characterised by slightly opposite behaviour also in other stress conditions. For example, the median of the group of "UPR Dependent Up" genes was slightly higher also in the hypo-osmotic shock conditions whereas the median of the "UPR Dependent Down" genes was lower in these conditions.

592 genes were assigned to groups "UPR Independent Up" and 603 "UPR Independent Down". These gene groups (Fig 1E, F), in contrast to UPR dependent gene groups, were effected also by many other types of stress. For example 105 of "UPR Dependent Up" and 305 of "UPR Dependent Down" group members are environmental stress genes, a form of general stress response defined by [17]. Genes assigned to groups 2, 7 and 8, described, provided and plotted in supplemental data, were judged to be outside the scope of this publication.

The content of the gene groups was compared also based on the number of genes in different function and location categories (Table 1). All gene groups up-regulated under secretion stress contained a higher percentage of secretion related genes compared to the total of the genome content of *S. cerevisiae*, whereas the down regulated gene groups showed a lower percentage of secretion related genes compared to the genome content (Table 1). Especially in the "coreUPR" group the secretion related genes were overrepresented, 63% of the genes vs. 26% of the genes in the whole genome. The second most prominent category was the genes related to transcription. In the gene group "UPR Dependent Up" the percentage of transcription related genes is well above their total amount in the genome, 26% vs. 17%. It is of interest that there is little evidence for down regulation of genes encoding secreted proteins in *S. cerevisiae *transcriptome data in secretion stress conditions (Table 1). In total, ten transcripts encoding proteins localised outside the cell showed down regulation independently of UPR, but this is only 15% of the genes encoding extracellular proteins in *S. cerevisiae *and the possible targets for the RESS phenomenon described in filamentous fungi [7,8].

### EST collection derived from cDNA-AFLP and cDNA subtraction library analysis of *T. reesei *cultures under secretion stress conditions

In order to identify genes that are differentially expressed in response to secretion stress in *T. reesei*, we prepared cDNA subtraction libraries from chemostat cultures of a tPA producing transformant of *T. reesei *Rut-C30 and its parental strain and from a batch bioreactor culture of *T. reesei *Rut-C30 treated with DTT and an untreated reference culture. In addition, the same cultures were subjected to cDNA-AFLP analysis to gather additional information using another method.

In preparation of the subtraction libraries, the sample and reference libraries were hybridised repeatedly to enrich fragments that are more abundant in the sample library. Progress of hybridisations was monitored by hybridising DNA probes such as *bip1 *and *pdi1 *gene fragments to dotplots of libraries and measuring the amount cDNA removed from libraries in each hybridisation cycle by radioactivity. Even after one round of hybridisation a clear selective enrichement of *bip1 *and *pdi1 *mRNA could be seen. In each hybridisation round some cDNA was removed indicating removal of non-regulated genes (data not shown). To cover broadly the produced libraries, four of those were selected for further analysis: libraries representing the culture treated with DTT for 60 min after 1 and 5 hybridisation cycles and libraries representing the tPA producing culture after 1 and 5 hybridisation cycles. In the cDNA-AFLP experiment the sample and reference cDNAs are amplified by a PCR based protocol and fractionated using electrophoresis. Bands that showed very clear up regulation in both (tPA and DTT 60 min) stressed samples were sequenced.

In total 2144 individual EST sequences were retrieved, including 94 fragments obtained from the cDNA-AFLP analysis. The ORFs corresponding to the ESTs were identified based on sequence similarity searches against the prepublication version of the *T. reesei *genome and the predicted *T. reesei *proteome, and subsequently the putative homologues of the corresponding ORFs were identified in the *S. cerevisiae *genome [20] and Swissprot [21]. Annotation of previously uncharacterised genes in *T. reesei *was done based on sequence similarity of the predicted ORFs of *S. cerevisiae*, since the *S. cerevisiae *genome annotation is much more advanced compared to other fungal species, and the UPR response of *S. cerevisiae *is characterised in detail. The EST sequences representing the tPA gene (98 sequences) were removed from the dataset before further analysis. Remaining 2047 sequences were submitted to EMBL with accession numbers AM111376 – AM113422. As multiple short ESTs can be produced for an individual gene by the methods used, the number of ESTs does not reveal the number of ORFs present in cDNA subtraction libraries or selected by cDNA-AFLP analysis. 1850 ESTs were found to correspond to 409 predicted ORFs in the genomic sequence. In addition 97 ESTs matched outside the predicted ORFs in the *T. reesei *genome version used and two of the ESTs matched to the mitochondrial genome published earlier [22]. These 97 sequences are likely to correspond to 48 ORFs. 99 sequences did not find a match in the currently available *T. reesei *sequences. These correspond to 71 unique sequences that remained completely unidentified.

Table 2 shows the 25 most abundant ORFs in the EST collection prepared from the tPA producer and from the DTT treated culture. These include *bip1 *and *pdi1*, expected to be up regulated under secretion stress conditions in *T. reesei *[5,7], and ORFs assigned to functions expected to be related to UPR in other organisms, such as *ubi4*, *ura8*, *itr2*, *prp2 *and *vps1 *(reviewed in [23,24]). Also transcripts expected to be very abundant, such as those encoding translation elongation factor *tef1 *[25], actin *act1 *[26], *hex1 *[27], cellobiohydrolases [28] and ribosomal genes [26] were represented in the libraries. These are likely to be false positives as for example northern analysis of *cbh1 *and *egl1 *shows that they are repressed in DTT treatment and tPA producing culture (data not shown).

Some differences were detected in the content of the libraries and in the abundance of ESTs corresponding to a particular gene. ESTs of some UPR related genes (*bip1*, *ubi4*, *pdi1 *and *vps1*) were more abundant in the library derived from the DTT treated culture compared to the library from the tPA cultivation, while some genes showed an opposite trend (*ura8*, *itr2 and erp38*). Only one cellobiohydrolase EST was found in the DTT treatment library while 119 were found in the tPA cultivation library. Half of ORFs in the libraries were represented only by a single EST preventing further analysis of significant differences between the libraries. Thus, combined content of the EST libraries is presented if not otherwise stated. A list of identified ORFs is provided in the supplemental data.

39% of the *T. reesei *ORFs found in the subtraction libraries or using cDNA-AFLP analysis and for which a putative function could be assigned were related to protein secretion (Table 1) which is well above the frequency of the appearance of secretion related genes in *S. cerevisiae *genome. The number of genes involved in transcription in the *T. reesei *EST collection was close to the relative amount of the transcription related genes that are up regulated in a UPR dependent manner in *S. cerevisiae *as defined by [2]. Both frequencies are lower than the frequency of transcription related genes in the *S. cerevisiae *genome. The more detailed dissection of the UPR up regulated genes of *S. cerevisiae *to the "coreUPR" genes and "UPR Dependent UP" in our study revealed a stronger segregation between the secretion related genes to the two groups, 63% of the "coreUPR" genes were secretion related whereas of the "UPR Dependent UP" only 31%. Also the frequency of genes involved in transcription differed in the two groups, only 7% of the genes in the group of coreUPR were transcription related genes, whereas 26% of the "UPR Dependent UP" genes were involved in transcription.

Interestingly, a more detailed comparison of the genes represented in the *T. reesei *EST set and the UPR dependently up-regulated genes revealed by our data analysis or by [2] showed relatively few homologous genes in common. However, no genomic scale data exists about functional homology relationships between *T. reesei *and *S. cerevisiae *genes to enable interpretation of this.

Our analysis of genes up-regulated in *T. reesei *under secretion stress conditions revealed also features that clearly differ from the ones observed in *S. cerevisiae*. The set of *T. reesei *ESTs representing putative secretion stress related genes contained many EST corresponding to *cpc1*, homolog of the *S. cerevisiae *gcn4, as well as three histone genes. Neither gcn4 nor histone genes were shown to be up regulated in our reanalysis of *S. cerevisiae *transcriptome data. Instead Gcn4p has been shown to be translationally induced [3]. Expression of *cpc1 *and histone genes under secretion stress in *T. reesei *was analysed in more detail as described below.

### Northern analysis of a selected set of genes corresponding to the ESTs revealed by the cDNA subtraction and cDNA-AFLP analysis

**Figure 2:**
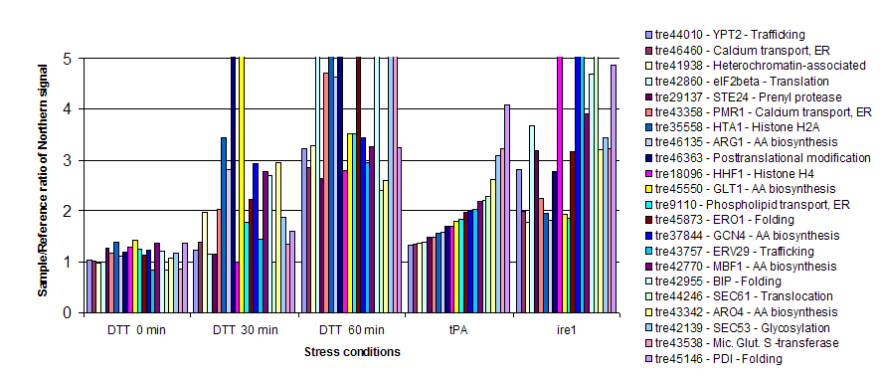
**Northern analysis of selected *T. reesei *genes revealed by the cDNA subtraction library and cDNA-AFLP analysis**. The expression level of the genes was analysed in cultures treated with DTT for 0, 30 and 60 min and in untreated reference cultures, in chemostat cultures of a tPA producing strain and its parental strain, and in a strain overexpressing *ire1 *and in its parental strain. The signals of the specific mRNAs were quantified and normalised using the signals of *gpd1*. The ratio of the signal in the analysed sample vs. the signal in a corresponding reference culture are shown for genes showing UPR-like gene expression. *T. reesei *protein model identifier (genome version 1.2), likely *S. cerevisiae *homologue and function or process in which the gene is involved is indicated after gene name. DTT 0 min, DTT treatment 0 min; DTT 30 min, DTT treatment 30 min; DTT 60 min, DTT treatment for 60 min; tPA, tPA producing chemostat culture; ire1, *ire1 *overexpressing strain.

To confirm the regulation of genes revealed by the cDNA subtraction and cDNA-AFLP analyses we first selected a random set of genes corresponding to ESTs and checked induction of the genes using Northern analysis. In the set of 23 genes, twelve genes showed more than 2-fold induction after one hour treatment with DTT indicating that about half of the unique genes of the EST library derived from the DTT treated culture are actually induced (data not shown). After the random initial set of Northern analyses, we chose to analyse expression levels of genes that could reveal essential new features of *T. reesei *secretion stress. 60 novel candidates for UPR related genes, based on the functional assignments of the homologous genes in other organisms were selected for the analysis. The genes *bip1 *and *pdi1 *known to be induced under these conditions were included in the study for comparison. Deletion strains of *T. reesei ire1 *or *hac1 *have not been obtained and could not be used in the analysis of UPR dependent gene expression in *T. reesei*. Therefore we defined genes showing UPR-like regulation as genes significantly induced (p < 0.016) after one hour treatment of the culture with DTT, as well as in culture of the tPA producing transformant and in culture of the *ire1 *overexpressing strain that shows constitutive activation of the regulator HAC1 and induction of its target genes [29]. However, this does not formally exclude the possibility that the induction of a certain gene could not be caused by a non-secretion stress specific pathway. By these criteria, 20 genes out of the set of 60 showed UPR-like regulation (Fig. 2). Nine of these genes belong to the categories previously described as UPR related ([2] or reviewed in [23,24]) such as genes involved in translocation, ER calcium homeostasis, folding, glycosylation, vesicle trafficking and cell wall biosynthesis. The remaining 11 genes revealed novel features in the secretion stress in *T. reesei*, and are described more in detail below.

### Nucleosomal genes are induced in secretion stress in *T. reesei*

As shown in Table 2, many ESTs corresponding to histone H2A gene are present in the secretion stress up-regulated EST collection, and based on Northern analysis, the genes for histone 2A and 4 are expressed in an UPR-like manner (Fig. 2). The H2A and H2B genes are closely linked in the *T. reesei *genome as are the H3 and H4 genes. The number and localisation of *T. reesei *histone ORFs resemble, but are not identical to the histone gene organisation of the filamentous fungi *Aspergillus nidulans *[30] and *Neurospora crassa *[31].

In order to elucidate whether the induction of histones in secretion stress is more widespread in filamentous fungi and not only limited to *T. reesei*, the expression level of the gene encoding the H4 core histone was analysed in a DTT treated culture of *A. nidulans*. The analysis showed a 2-fold induction of the gene between 30 minutes and 120 minutes of DTT treatment (Fig. 4).

### CPC1/GCN4 is induced in *T. reesei *and *A. nidulans *secretion stress

ESTs corresponding to the *T. reesei *homologue of *cpc1 *(cross pathway control) in *N. crassa *(*GCN4*, general control nitrogen, in *S. cerevisiae*) are abundant both in the subtraction library derived from the DTT treated culture as well as in the one derived from the tPA producing culture (Table 2). Based on Northern analysis, the expression level of the gene was induced in the DTT treated culture as well as in cultures producing tPA or overexpressing *ire1*, thus indicating UPR-like regulation (Fig. 2).

Also the homologue for the *S. cerevisiae MBF1 *was isolated from the subtraction libraries and was among the UPR induced genes (Fig. 2). *S. cerevisiae *Mbf1p is a transcriptional co-activator, that has been shown to mediate Gcn4p dependent transcriptional activation by bridging the DNA binding region of Gcn4p and Spt15p (TATA-binding protein) [32]. However, *MBF1 *has not been described as a Gcn4p target [33-35]. The secretion stress up-regulated EST collection contained also several ESTs corresponding to putative homologues of *S. cerevisiae *SPT15 and TAF2 (TATA binding protein-Associated Factor).

**Figure 3:**
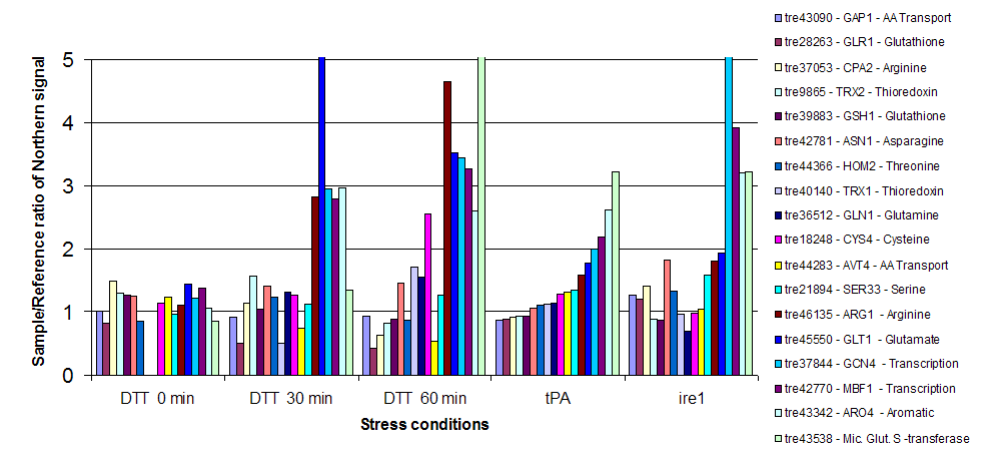
**Northern analysis of genes related to amino acid biosynthesis and glutathione metabolism in *T. reesei***. The expression level of the genes was analysed in bioreactor cultures treated with DTT for 0, 30 and 60 min and in untreated reference cultures, in chemostat cultures of a tPA producing strain and its parental strain, and in a strain overexpressing *ire1 *and in its parental strain. The signals of the specific mRNAs were quantified and normalised using the signals of *gpd1*. The ratio of the signal in the analysed sample vs. the signal in a corresponding reference culture are shown. *T. reesei *protein model identifier (genome version 1.2), likely *S. cerevisiae *homologue and amino acid for whose biosynthesis or use the gene is involved is indicated after the gene name. DTT 0 min, DTT treatment 0 min; DTT 30 min, DTT treatment 30 min; DTT 60 min, DTT treatment for 60 min; tPA, tPA producing chemostat culture; ire1, *ire1 *overexpressing strain, AA transport, amino acid transport.

To study expression of *T. reesei cpc1 *target genes under secretion stress conditions, putative candidates for CPCI targets in the *T. reesei *prepublication version of the genome were identified based on sequence similarity searches against predicted *S. cerevisiae *Gcn4p targets [33-35]. Among these we selected genes that were represented in the EST collection or that were available as cloned ESTs from [36]. Additionally we selected *trx2*, *trx1*, *gsr1 *(a homologue of S. cerevisiae *glr1*), and *gsh1 *due to their role in glutathione metabolism. Northern analysis of the genes under secretion stress conditions was carried out (Fig. 3). Of the selected set of genes, *glt1 *(glutamate synthase), *arg1 *(arginosuccinate synthetase) and *aro1 *(pentafunctional arom protein, aromatic aminoacid biosynthesis) showed UPR-like regulation. Also *asn1 *(asparagine synthetase) was induced almost 2-fold in the *ire1 *over-expressing strain and *cys4 *(cystathionine beta-synthase) was more than 2.5-fold induced after one hour of DTT treatment, but the genes were induced to a lesser extent in the other conditions studied.

The glutathione S-transferases (GST) are a family of enzymes involved in the detoxification of oxygen radicals and other reactive intermediates (reviewed in [37]). A putative homologue of a human microsomal glutathione s-transferase 3 gene (MGST3) was found among the ESTs representing genes up-regulated under secretion stress. UPR-like regulation of the gene in *T. reesei *was confirmed using Northern analysis (Fig. 2).

**Figure 4:**
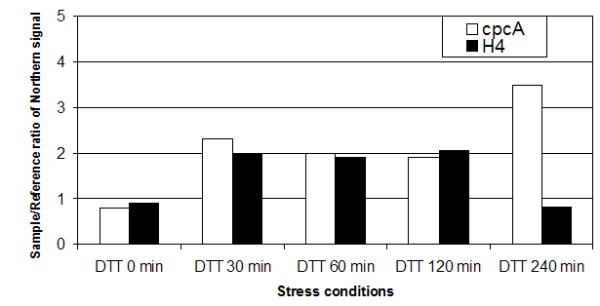
**Northern analysis of A. nidulans *cpcA *and histone H4 genes in a culture treated with DTT and in an untreated reference culture**. The expression level of the genes was analysed in a culture treated with DTT for 0, 30, 60, 120 and 240 min and in untreated reference culture. The signals of the specific mRNAs were quantified and normalised using the signals of *gpd1*. The ratio of the signal in the analysed sample vs. the signal in a corresponding reference culture are shown. cpcA [70], H4 [30].

The induction of *cpc1 *in secretion stress was confirmed also to take place in the DTT treated culture of *A. nidulans*. The gene cpcA was induced 2-fold after 30 min treatment with DTT (Fig. 4).

### Amino acid biosynthesis genes seem not to be induced in *S. cerevisiae *by secretion stress

In contrast to our finding in *T. reesei*, the GCN4 of *S. cerevisiae *has not been shown to be induced under secretion stress [2,3,17-19]. However, activation of Gcn4p synthesis has been reported under tunicamycin treatment in *S. cerevisiae *[3]. As secretion stress in *T. reesei *resulted in induction of also a set of putative target genes of the GCN4 homologue CPCI, including some amino acid biosynthesis genes, we compared the expression of the putative Gcn4p targets in the transcriptome profiling data set combined from literature.

**Figure 5:**
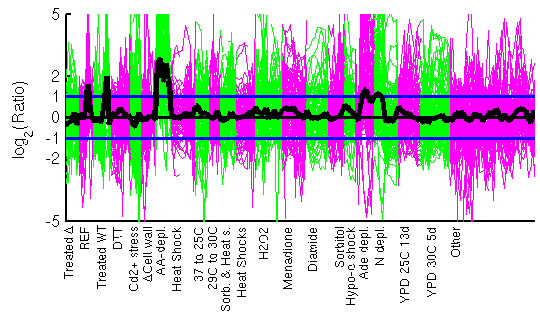
**Expression of *S. cerevisiae *amino acid biosynthesis genes controlled by GCN4 in response to amino acid deprivation from [35] in 174 different stress experiments assembled from various articles**. The expression levels are shown as log_2 _of signal ratios stressed/reference cultures in 174 different stress experiments assembled from various articles [2, 3, 17, 18, 35, 48, 49]. The expression values of individual genes as **purple**and **green**striped lines, the median of gene group in **black**, standard deviation of the whole experiment in **blue**. Experiment sets are explained in Fig 1.

Fig. 5 shows expression data on the amino acid biosynthesis genes that have been indicated as Gcn4p targets based on transcriptional profiling data on *S. cerevisiae *strains with constitutive GCN4 expression or GCN4 deletion [35]. These genes are clearly induced in amino acid or nitrogen depletion conditions [17,35], the median gene expression value of the gene set showing 2-fold induction. In secretion stress conditions (Fig. 5; the data sets "Treated WT", "Treated Δ", "REF") the median log_2 _ratio of the gene set is 0, except in one reference experiment ("REF" data set) and in tunicamycin treatment ("Treated WT" data set), both derived from data by [18]. The Gcn4p target gene sets selected based on computational predictions [33,34] show the same result in the expression profiling data although their response to amino acid depletion is less pronounced (data not shown).

### No clear lack of amino acids can be detected from *T. reesei *cells under secretion stress

As *cpc *genes are known to be induced transcriptionally in response to amino acid deprivation in filamentous fungi [38,39], we wanted to determine whether there was such a lack in *T. reesei *in our culture conditions. The concentrations of total intracellular free amino acids were determined from tPA producing strain, a DTT treated culture and culture of *ire1 *over expression strain and their respective reference cultivations by HPLC. Of the 20 standard amino acids 15 were determined successfully. No systematic decrease of concentration of a single amino acid in all cultivations could be seen. Instead in DTT treated culture after 120 min the concentrations of alanine, arginine, and threonine were at 2.9, 3.2, 2.1, fold higher level (average of two measurements, respectively for alanine, arginine and threonine) compared to the reference culture.

## Discussion

### Computational reanalysis of published *S. cerevisiae *transcriptome profiling data allows the dissection of the effects of secretion stress

*S. cerevisiae *has served as one of the model organisms in studies on protein secretion and on factors affecting the process. The mechanisms of the unfolded protein response (UPR) activated under conditions where protein folding and transport is hampered is well characterised in *S. cerevisiae*, and transcriptional profiling data on the responses to impaired secretion process i.e. secretion stress inducing conditions, is available from various sources [2,3,17-19]. In addition there is data on responses to a large variety of other type of stress conditions. In order to create a consensus model of the responses to secretion stress in *S. cerevisiae *and to enable comparison of the corresponding responses in other organisms, we have combined the transcriptional profiling data currently available in literature. In comparison to previous studies of transcriptomic effects of secretion stress, we used a larger and more varied data set as it was combined from all the previous studies. This enables exclusion of effects specific to inducer of stress (e.g. DTT, tunicamycin or heterologous protein), the specific amount of chemical or the strain used. We also provide lists of genes upregulated or downregulated, either in UPR dependent or independent manner. This computational analysis provided a robust dissection of transcriptional effects of secretion stress in *S. cerevisiae *to compare our results of transcriptional effects of secretion stress in *T. reesei*.

Our analysis of the combined data on secretion stress in *S. cerevisiae *revealed eight specific groups of genes showing differential expression under the secretion stress conditions. Namely, the model selected a group of 46 strongly responding UPR-dependent genes ("coreUPR"), that were mainly up-regulated under the secretion stress conditions and not as a response to other type of stresses, and another group of 135 UPR-dependent up-regulated genes showing less stringent response to secretion stress (Fig. 1A and 1C). Both groups, but especially the "coreUPR" group, had a higher content of secretion related genes compared to the relative amount of secretion related genes in the genome, which is in accordance with previous information on the functional categories of UPR up-regulated genes [2,23,24]. [2] previously identified a set of 381 genes induced in a UPR dependent manner (Fig. 1, panel B) based on their similar behaviour to predefined model UPR genes (Fig. 1, panel B, shown in yellow) in transcriptional profiling experiment. Our test showed only 86 of these genes to be UPR dependently induced (35 were classified into "coreUPR" group, 51 in "UPR Dependent Up" group). The analysis revealed also a large number of genes that were up regulated (592 genes, "UPR Independent Up") or down regulated (603 genes, "UPR Independent Down") under secretion stress conditions, but not in a UPR dependent manner that would require functional IRE1 and HAC1 genes. 60 genes of the UPR related genes from [2] were assigned to "UPR Independent Up". The genes in these groups were responsive also to other type of stress conditions (Fig. 1E and 1F), indicating that they are regulated probably through general stress pathways.

*T. reesei*, *Aspergillus niger *and *Aspergillus nidulans*, exhibit transcriptional down regulation of secreted proteins in secretion stress (REpression under Secretion Stress, RESS) [7,8,40]. It has been also shown in plant cultures (*Arabidopsis thaliana*) that many of the genes encoding proteins with putative signal sequences are down-regulated during treatment with DTT or tunicamycin [9]. For *S. cerevisiae *this has not been reported, and as shown by the trancriptome profiling studies for *S. cerevisiae *secretion stress [2,17-19] and by our computational analysis (Table 1), only a few secreted proteins show down regulation in their transcript amounts under secretion stress in *S. cerevisiae*. As the transcriptomic responses of *A. nidulans *[40] and in *A. thaliana *[9] appear to affect larger amount of genes encoding secreted proteins, it is very likely that *S. cerevisiae *does not have the RESS response.

### Analysis of the EST collection reveals novel aspects of secretion stress in *T. reesei*

Transcriptomic responses in *T. reesei *secretion stress were studied under different conditions provoking secretion stress. cDNA subtraction library and cDNA-AFLP analyses were carried out from cultures producing a heterologous protein, tPA, and from cultures treated with DTT to prevent protein transport and folding. Furthermore, cultures of a strain overexpressing the UPR pathway regulator gene *ire1 *were included in a further study for confirmation of induction of a selected set of genes under secretion stress using Northern analysis. The results from the combined analysis of the EST collection of different conditions confirmed the published results on the induction of previously characterised UPR related genes, such as of *bip1 *and *pdi1 *[5,7] (Table 2). Furthermore, ESTs corresponding to at least 457 genes putatively involved in secretion stress in *T. reesei *were isolated. The EST collection contained genes from functional categories expected to be up regulated under secretion stress conditions based on transcriptomic studies in *A. thaliana*, *A. nidulans *and *S. cerevisiae *[2,9,40] and it was clearly enriched in the number of secretion related genes as compared to the genome content of secretion related genes of *S. cerevisiae *(Table 1).

The responses induced by production of a heterologous protein and by the DTT treatment might also have features specific to each of these two conditions and therefore we have focused on genes under differential expression in all culture conditions. A gene was classified as a UPR-like gene if it was confirmed by Northern analysis to be up regulated after one-hour-treatment with DTT, in a tPA producing culture as well as in a strain overexpressing *ire1*. Northern analysis of a set of genes selected from the EST collection revealed UPR-like up regulation of a group of genes expected to be UPR up-regulated based on data obtained from other organisms, e.g. *bip1*, *pdi1*, *ero1, pmr1 *and *sec61 *or belonging to functional categories UPR up-regulated in other organisms *sec53*, *ypt2*, *ste24*, *erv29 *[2,9,40] (Fig. 2). Many of the genes analysed by Northern analysis were induced at a lower level in the tPA producing culture than in the DTT treated culture or in the strain overexpressing *ire1 *(Fig. 2). This was reflected also in the abundance of ESTs corresponding to the differentially expressed genes in the different libraries, as demonstrated by the abundantly expressed foldase/chaperon genes *pdi1 *and *bip1 *(Table 2). In addition, the analysis revealed induction of genes that have not been described as UPR induced in filamentous fungi or yeast, which are discussed more in detail below.

### Nucleosomal genes were induced as a response to secretion stress in *T. reesei *indicating uncoupling of their regulation from cell cycle control

The core histones are generally thought to be strictly regulated with cell cycle, active synthesis taking place during the S-phase [41,42]. *S. cerevisiae *transcriptome data collected for this study indicate that if some regulation of nucleosomal genes as a response to stress is taking place, it is primarily down regulation, and no up regulation is seen under secretion stress conditions (data not shown). On the contrary, the histone genes H2A and H4 were induced in *T. reesei *secretion stress in a UPR-like manner (Fig. 2). A transient induction of H4 histone gene was also observed in cultures of *A nidulans *subjected to DTT treatment (Fig. 4), indicating that the nucleasomal induction could be a more common phenomenon in the filamentous fungi. The induction of H2A and H4 appears to have no correlation with growth rate of the cells. The comparison of the expression levels of the genes in the tPA producing strain and in its parental reference strain was carried out in well controlled chemostat cultures with the same specific growth rate. Growth activation could not explain the induction either, since the treatment of the cultures with DTT retards the growth to some extent, and also the *ire1 *overexpression does not lead to increased growth of the fungus. Further studies are required to elucidate the significance of the increased histone gene expression under conditions where no obvious increase in DNA synthesis takes place.

### *cpc1 *induction of *T. reesei *resembles ATF4 controlled secretion stress response in mice

The *T. reesei *EST collection of genes upregulated under secretion stress contained many ESTs homologous to cpc genes of filamentous fungi or to the counterpart in *S. cerevisiae*, GCN4 (Table 2). UPR-like regulation of the gene in *T. reesei *was confirmed by Northern analysis (Fig. 2). The CPC proteins in filamentous fungi as well as the *S. cerevisiae *GCN4 have been shown to control amino acid biosynthesis [38,39]. GCN4 is thought to be involved also in responses to purine starvation, glucose limitation, growth on ethanol, high salinity, treatment with methyl methanesulfonate and treatment with rapamycin (for review see [43]). A notable difference in activation of the CPC homologues and GCN4 in response to amino acid deprivation is that *S. cerevisiae *GCN4 is mostly regulated on translational level [44] whereas the CPC-proteins in filamentous fungi are also under strong transcriptional control [38,39]. The *S. cerevisiae Gcn4p *and the mouse homologue ATF4 have also been shown to be involved in secretion stress [3,12,13]. In *S. cerevisiae*, Gcn4p is required for induction of the majority of UPR induced genes under secretion stress [2]. Mouse Atf4p appears to transcriptionally induce genes involved in glutathione biosynthesis under tunicamycin treatment. It has been suggested based on a variety of functional assays that the lack of reducing power and the need to up regulate glutathione biosynthesis were the major reasons for ATF4 induction in mouse under secretion stress conditions [13].

We carried out a Northern analysis in secretion stress conditions of a set of *T. reesei *genes whose homologues in *S. cerevisiae *have been shown to be Gcn4p targets based either on computational promoter analysis [33,34] or microarray experiments [35]. As the role of CPC proteins in regulation of amino acid biosynthesis is very similar in *S. cerevisiae *and in filamentous fungi, it is likely that many of these selected genes are *cpc1 *targets also in *T. reesei*. The genes *glt1*, *arg1 *and *aro1 *were shown to be UPR induced genes in *T. reesei *based on the Northern analysis. In addition, the gene *asn1 *was induced almost 2-fold in the *ire1 *over-expressing strain and *cys4 *was induced more than 2.5-fold after one hour DTT treatment (Fig. 2 and 3). In mice a homologue of *asn1*, asparagine synthase (Asns) and a homologue of *cys3*, cystathione γ-lyase (Cth) which follows *cys4 *in homocysteine and cysteine interconversion – pathway, show an ATF4 dependent regulation in secretion stress [13]. The glutathione biosynthesis gene *gsh1*, thioredoxin genes (*trx1*, *trx2*) and glutathione reductase (*gsr1*) involved in glutathione related functions do not show up regulation in *T. reesei*, which is also the case for their homologues in mouse [13]. GLNI that synthesises glutamine from glutamate instead of glutathione neither shows any induction. At transcript level our results with *T. reesei *are in accordance with the observations in mouse in that not all the genes putatively under ATF4 regulation or involved in glutathione metabolism are affected under secretion stress conditions.

In mice, glutathione-s-transferases are activated to protect cells from oxidative stress [45] and in ER stress conditions [46]. The homologue of human microsomal glutathione s-transferase 3 (MGST3) was also among the UPR activated genes in *T. reesei *(Fig. 2). In order to determine whether induction of *cpc1 *in *T. reesei *could be due to amino acid starvation, we determined intracellular total concentrations of 15 amino acids. No systematic lack of any of these amino acids in all conditions was detected. Based on the assembled evidence we believe that *T. reesei *is manifesting a response similar to the ATF4 dependent response of mouse.

## Conclusion

We have isolated a rather large variety of genes as putative candidates for secretion stress related genes in *T. reesei*. Further analysis of the genes induced under secretion stress has revealed novel features in the stress response in *T. reesei *and in filamentous fungi. We have demonstrated that in addition to the previously rather well characterised induction of genes for many ER proteins or secretion related proteins also other types of responses exist. Nucleosome genes were induced both in *T. reesei *and in *A. nidulans*, this response has not previously been found from other organisms. Furthermore our results suggest that the response to secretion stress in *T. reesei *has similarities to mammalian cells in the role of *cpc1*/ATF4, which has been suggested to enhance, together with a subset of its target genes, glutathione synthesis and to alleviate oxidative stress in the ER. Further studies are needed to fully uncover the role of *cpc1 *in secretion stress in *T. reesei *and to elucidate whether the regulatory mechanisms of UPR involve also CPCI as a direct transcriptional factor involved in activation of UPR target genes in a similar manner as the Gcn4p in *S. cerevisiae *[3].

## Methods

The transition from pre-genomic to post-genomic era in *T. reesei *research took place during the preparation of this publication. The work was planned and most laboratory experiments executed without any genomic data, but while carrying out Northern and data analysis we used *T. reesei *QM6a version 1.0/1.2 genome provided by Joint Genome Institute [47].

### Comparison of *S. cerevisiae *transcriptome profiling data

Transcriptome profiling data from *S. cerevisiae *at different conditions was obtained from literature [2,3,17-19,35,48,49]. The outlier removal and normalisations provided by the authors in the original articles were used. Repeats of the same experiment were averaged and all measurements were transformed into log_2 _of the ratio of the signals under stressed conditions to those under reference conditions. Frequency distributions were visually checked and acceptable data was median centred and variance normalised. The response of an individual gene to secretion stress was defined as follows. To define *IRE1 *and *HAC1 *dependent reaction to secretion stress, values in response to production of a heterologous protein and to tunicamycin treatment [19], to 60 min DTT treatment [17], to 60 min tunicamycin and DTT treatment [2], to 30 min DTT treatment [3] and tunicamycin treatment [18] of wild type strains were used as samples (named as an experiment set "Treated WT"), and compared to the values obtained from Δ*IRE1 *Δ*HAC1 *deletion strain in 60 min tunicamycin or DTT treatments [2] and from Δ*IRE1 *strain in 30 min DTT treatments [3] (defined as an experiment set "Treated Δ"). To define *IRE1 *and *HAC1 *independent reaction to secretion stress the same sample values were compared to Heat Shock 000 minutes, 0 min DTT and 5 min DTT treatment [17] and two randomly picked reference experiments (ds800 vs. ds799 and ca1412 vs. ca1411 as described in [18] (named as an experiment set: reference, "REF"), where no responses apart from technical variation should be seen. Only those genes with data from all these experiments were accepted (5810 genes for dependent and 4697 for independent was taken into account). The values of each gene within the defined experiment sets ("Treated WT", "Treated Δ" and "REF") were treated as repeats of the same phenomena. The significance of the differences in the values between the experiment sets were evaluated with a Wilcoxon ranksum test [50] using a cut-off of p < 0.05, and genes showing significant difference were divided into eight gene groups. Five of them (Groups 1,3,4,5 and 6) are presented here and the rest with their selection rules are provided as supplemental data [see Additional file 1 and 2]. The rules below were used to pick groups 1 to 6. The rules were applied in sequence of the group numbers, for example genes of group 1 would fill the requirements of group 3 and 5, but only belong to group 1.

Group 1 ("coreUPR"), both tests (difference between "Treated WT" and "REF" and difference between "treated WT" and "Treated Δ") significant and median_treated WT _> median_REF _and median_treated Δ _≤ median_REF_;

Group 2 ("coreUPR Down"), both tests tests (difference between "Treated WT" and "REF" and difference between "treated WT" and "Treated Δ") significant and median_treated WT _< median_REF _and median_treated Δ _≥ median_REF_;

Group 3 ("UPR Dependent Up" regulation), significant difference between "Treated Δ" and "Treated WT" and median_treated WT _> median_REF _and median_treated Δ _≤ median_REF _and not already assigned to group 1;

Group 4 ("UPR Dependent Down" regulation), significant difference between "Treated Δ" and "Treated WT" and median_treated WT _< median_REF _and median_treated Δ _≥ median_REF _and not already assigned to group 2;

Group 5 ("UPR Independent Up" regulation), significant difference between "Treated WT" and "REF" and median_treated WT _> median_REF _or both tests (difference between "Treated WT" and "REF" and difference between "Treated WT" and "Treated Δ") significant and median_treated WT _> median_REF _and median_treated Δ _> median_REF _and not already assigned to group 1 or 3;

Group 6 ("UPR Independent Down" regulation), significant difference between "Treated WT" and "REF" and median_treated WT _< median_REF _or both tests (difference between "Treated WT" and "REF" and difference between "Treated WT" and "Treated Δ") significant and median_treated WT _< median_REF _and median_treated Δ _< median_REF _and not already assigned to group 2 or 4;

To describe the content of the gene groups, the amount of genes corresponding to certain functional and localisation categories from CYGD (FunCat version 2.0) [51] was counted.

### Strains and culture conditions

*Trichoderma reesei *Rut-C30 [52] transformed with the plasmid pJMU306 to generate strain *T. reesei *306/36 was kindly provided by J. Uusitalo (VTT Biotechnology). The plasmid pJMU306 contains the catalytic and linker regions of *T. reesei *cellobiohydrolase 1 (cbh1) gene fused with the coding region of human tPA gene [53]. A Kex2 cleavage site was inserted between the CBHI and tPA encoding regions and a sequence encoding (His)6 tag was located in the C-terminus of the construct following the tPA encoding region. The cbh1 promoter and terminator were used for expression of the fusion protein (J. Uusitalo, personal communication)

Pre-cultures for *T. reesei *bioreactor cultivations were carried out in 40 ml of minimal medium containing lactose (8 g/l) as a carbon source in 250 ml Erlenmeyer or Nephlos flasks [54]. The medium for the shake flask cultures was also supplemented with 1.5 g/l Stabileze QM (Methyl Vinyl Ether/Maleic Anhydride copolymer crosslinked with 1,9 Decadien, ISP Technologies, Inc.) or Junlon [55]. The flasks were inoculated with 106 spores ml^-1^. Cultures were incubated on a rotary shaker at 200 rpm (throw = 2.5 cm) at 30°C until used as an inoculum for batch and continuous bioreactor cultures.

Cultivation for drug treatment (10 mM DTT) was carried out in an Applikon (FT Applikon Ltd., Tewkesbury, UK) bioreactor (2.3 l full working volume) on the minimal medium with 20 g/l lactose as the carbon source. After growth to mid-exponential phase, the cultures were split in two halves, the other half was used as reference (no DTT added) and the other half was treated with 10 mM DTT. Samples were taken regularly up to 360 minutes after drug treatment from both the reference and the treated culture.

Chemostat cultures were carried out in an Applikon (FT Applikon Ltd., Tewkesbury, UK) bioreactor (2.3 l full working volume) according to the methods of [56] using minimal medium containing 8 g/l lactose as a carbon source. The cultures were inoculated with 200 ml pre-culture, grown in minimal medium with 10 g/l lactose for 3 days. The Cultures were maintained at 28°C ± 1°C and pH 5.5 ± 0.1, agitated at 900 – 1000 rpm with a 3 six-bladed (48 mm diameter) Rushton turbine impeller. Aeration in the cultures was set to approx. 0.7 vvm (l air [l culture]-1 min-1). Foaming was controlled by continuous addition of a mixture of polypropylene glycol (PPG) of different molecular weights PPG 1025 (BDH): PPG 2025 (BDH): FoamMaster PPG (mixed molecular weight; Henkel Performance Chemicals, Leeds, UK) in the ratio 2:2:1 (35) to give a final concentration of approximately 0.01% (v/v) PPG. The dilution rate was kept constant at 0.05 ± 0.005 h^-1^. In order to reduce the attachment of biomass to the surfaces inside the bioreactor, the impeller speed was increased to 1500 rpm once a day for about 15 minutes, after the daily sample had been taken.

A *T. reesei *Rut-C30 strain overexpressing *ire1 *[29] and its parental strain were grown in shake flasks for 3 days in minimal medium [57] with 3% lactose. *Aspergillus nidulans *FGSC A26 (biA1) was grown and treated with DTT as in [5].

### Analysis of growth and protein production in the bioreactor cultures

CO_2 _was analysed using an ADC 7000 infrared Gas Analyzer; (The Analytical Development Co. Ltd.; U.K). Biomass from bioreactor cultures (internal and overflow spill) was determined by filtering 2 × 10 ml of culture, samples from the bottom and the top of the bioreactor vessel, through pre-dried and pre-weighted Whatman No. 1 filter papers. The harvested biomass was washed with at least 20 ml of deionised water and dried to constant weight [70°C for at least 3 days or 30 minutes in a microwave at 260 watt]. Viable counts were determined as described in [58] to determine the combined fragment and conidial concentration in the culture, and also to be used as a determinant of fungal contamination.

Culture supernatant was obtained by filtration through 0.22 μm MILLEX^®^-GP syringe filters (Millipore Corporation, Bedford, MA, USA). Aliquots of the supernatant were stored frozen at -20°C for subsequent analysis. Total protein was determined by the assay of Bradford [59] using cellulase (EC 3.2.1.4) from Trichoderma reesei (Sigma C-8546) as the protein standard. Total cellulase activity (CBH1 and EG1) was determined essentially as decribed by [60], but using using p-nitrophenol-β-D-lactopyranosid (1 g/l, SIGMA N-1752) as substrate. The substrate (400 μl, 1 mg/ml in 0.05 M citric acid buffer, pH 5.0) was incubated with 50 μl culture supernatant (diluted as necessary) or standard (500 mg/l – 10 mg/l) in an Eppendorf tube for 1 h at 37°C. The reaction was stopped by adding 500 μl Na_2_CO_3 _(1 M) or Borax (0.1 M); Borax was used when addition of Na_2_CO_3 _caused precipitants to form. The absorbance of the p-nitrophenol released during the reaction was measured on a spectrophotometer at a wavelength of 400 nm. β-glucosidase and β-galactosidase activity were measured following the above protocol, but using p-nitrophenol-β-D-glucopyranosid (β-glucosidase) and p-nitrophenol-β-D-galactopyranosid (β-galactosidase) as substrates [61]. References to determine the absorbance contributed by the medium, were prepared by adding 500 μl Na_2_CO_3 _(1 M) or Borax (0.1 M) prior to addition of the sample or standard. The absorbance of the reference was subtracted from the reaction absorbance, prior to determining the enzyme concentration. Citric acid buffer (50 μl) with substrate (400 μl) and 500 μl Na_2_CO_3 _(1 M) or Borax (0.1 M) was used as a blank. Total tPA was determined using an ELISA-assay (INNOGENETICS N.V. Ghent, Belgium), according to the manufacturers' instructions, except the second incubation step was carried out for 4 hours at 4°C instead of 1 hour at 37°C.

### RNA extraction

Total RNA was isolated using the TRIZOL reagent (Gibco-BRL) as instructed by the manufacturer. For cDNA-subtraction libraries and cDNA-AFLP, poly-A fractions were isolated from total RNA with the Oligotex kit (Qiagen). The poly-A fraction's purity and quantity was determined by mRNA-Nano 6000 kit in the Agilent Bioanalyzer (Agilent).

### cDNA subtraction libraries

We used a modified protocol of the PCR-Based Subtractive cDNA Cloning [62]. RNA was extracted from the chemostat cultures of the tPA expressing strain and its parental strain *T. reesei *Rut-C30 and from the DTT treated culture and the untreated reference culture of Rut-C30 after 60 minutes of DTT addition. Double stranded cDNA (dscDNA) was synthesized from RNA poly-A fractions using SuperScript Double Stranded Synthesis Kit (Life Technologies). The dscDNA was digested with AluI and Rsa I enzymes (New England Biolabs). Adaptors for fragmented cDNA were annealed from following oligos (Sigma) and ligated to cDNA: for the stressed conditions (either the tPA producing culture or the DTT treated culture) A: 5'-GAGTATCAAGGATCCAAGCAT-3' and B: 5'-ATGCTTGGATCCTTGATACTCTTCA-3' and for the reference conditions (either the chemostat culture of the parental strain or the untreated Rut-C30 batch bioreactor culture) C: 5'-CTACATGCGTCTTAAGTTGAT-3' and D: 5'-GAGTACCAAGATATCCAGCAT-3'. The adaptor ligated cDNA was purified using Qiaquick Gel Extraction Kit (Qiagen). PCR was done from the purified cDNA with the adaptor oligos A or C for their respective fragment pools. Dynazyme EXT (Finnzymes) polymerase was used in all PCR reactions and PCR was carried out for 18 cycles with an annealing temperature of 54°C. Instead of biotinylated nucleotides, 5' biotinylated primers were used for preparing substracting cDNA. The PCR products were purified with Qiaquick Gel Extraction Kit (Qiagen). The PCR products from the stressed cultures were hybridised with the products from the corresponding reference cultures. Hybridisations were carried with 10 μg of the substracting cDNA and approximately 1 μg of the cDNA pool from which the unique cDNAs were to be isolated. Biotin labelled hybrids were removed with Dynabeads (Dynal) according to manufacturer's instructions. Repeated cycles of hybridisation were carried out and the progress of subtraction was monitored by scintillation counting of the hybridisation products, agarose gel analysis of PCR products and dotblots hybridised with relevant probes.

The subtracted cDNA fragment pools representing genes up-regulated in stressed conditions were digested with BamHI (the site located in the A/B adaptor) and cloned to pBK-MCV phagemid vector in XL1-Blue MRF' strain with ZAP Express Predigested Gigapack Cloning Kits (Stratagen). Colonies of XL1-Blue MRF' were picked with the automated colony picker QPix (Genetix). Plasmids were purified with Plasmid miniprep96 kit (Millipore) and sequenced with T7 and/or T3 primers with BigDye 1.1 kit in an ABIprism 9600 sequencer (Applied Biosystems) according to manufacturer's instructions.

### cDNA-AFLP

cDNA-AFLP experiments were carried out as described previously [63]. RNA was extracted from cultivation of the tPA expressing strain in a chemostat and the DTT treated cultivation after 60 minutes of DTT addition and their respective references. In short dscDNA was made from reference and stressed RNAs and respective pools of unique gene specific 3' fragments were made, amplified by PCR and fractionated using electrophoresis.

To generate the fragments, dscDNA were digested first with BstYI and then with MseI. For preamplification, BstYI end primers having a C in 3' end were used. A selection of three nucleotides was used, BstYI end with two selective nucleotides and MseI end with one selective nucleotide. Bands were scored visually and those that showed very clear up regulation in both stressed samples were cut out and sequenced.

### Northern analysis

Northern analysis was carried out using standard protocols [64]. ESTs from the cDNA subtraction library clones were PCR amplified using T7 and T3 primers, and the fragments were digested with BamHI to remove extra sequence and used as probes in the Northern analyses. Probes specific for the genes *gln1 *(CB896216.1), *cys4 *(CB895493.1), *ans1 *(CB904095.1), *glt1 *(CB898755.1), *aro4 *(CB903024.1), *hom2 *(CB902136.1) and *ser33*, (CB899553.1) were obtained by PCR amplification of clones from EST library [36] using the respective sequencing primers. Gene specific probe fragments from genomic DNA were PCR amplified using the following primers: A. nidulans H4 gene, gcgagagatgttgagaatgga and gtgaagcagttgggagacg; *cpcA *gtccacctgtcccgctc and atgtctccctgtcgctcaag; *T. reesei *microsomal glutathione-S transferase, tcccttcgtctctaccaacaam and ttgaggttcatttccatttcg; *gsr1*, taagacggagggtgtggaag and, gccgcaggaaggtgttgt; *trx2*, tgccgaagagttcaaaaagg and cagctcgtccacgtcaaa; *gsh1*, ttcactcaccccttttaccc and atgttttcgtccaccttctt; *trx1*, actggcaccatccaccac and gcctcctcgaccctcttct. All PCR amplified products were subsequently analysed on agarose gels, and purified using Qiaquick Gel Extraction Kit (Qiagen).

5 μg of total RNA isolated from the chemostat cultures of the tPA producing transformant and its parental strain and from the DTT treated culture and the corresponding untreated culture at different time points, as well as RNA isolated from the shake flask cultures of *ire1 *overproducing strain and its parental strain were subjected to the analysis. The Northern signals were quantified using a phosphoimager (Typhoon 8600, AmershamBiosciences). The log_2 _of the ratio of the signal in the stressed culture and in the reference culture were determined. The log_2 _ratio from DTT treatment after one hour (DTT treated culture vs. the untreated culture at the same time point), tPA producing transformant (tPA producer vs. the parental strain) and *ire1 *overproducing transformant (*ire1 *overproducer vs. its parental strain at the same time point) were treated as repeats and the significance of the values was tested against all log_2 _ratios in the DTT treated cultures just before addition of DTT. The significance of the quantified signals was evaluated by a Wilcoxon ranksum test [50] with a cutoff of p < 0,016 (lowest available p-value with this sample size).

### Sequence analysis

Basecalling was done with Phred [65]. Removal of low quality sequences, quality trimming, primer removal and removal of host genome sequences was done with the program Staden [66]. Smith-Waterman sequence similarity searches were carried out with Genematcher2 (Paracel) at Finnish IT centre for science. The sequences were matched to their corresponding predicted open reading frames in *T. reesei *genome [47] with a cut-off of 96% identity. Sequences not giving a reasonable match in *T. reesei *genome were matched against *Neurospora crassa *genome [67] and Swissprot [21]. Because the *T. reesei *annotation was incomplete the genes were placed in functional categories based on their *S. cerevisiae *[20] and Swissprot [21] sequence homologies. A cut-off of E < 1 × 10^-20 ^and a fixed database size of 9 × 10^9 ^(which was the approximate size off EMBL at the time the work was started) was used. Results of genomic matches were manually checked against the Swissprot [21] matches to make sure that none contradicted. tPA derived ESTs were removed based on sequence similarity prior to database searches. The homologies of the ESTs are provided in supplemental data [see Additional file 3]. In addition the amount of ESTs corresponding to certain functional and localisation categories from CYGD (FunCat version 2.0) [51] was counted.

### Determination of concentration of intracellular amino acids by HPLC

The amino acid concentrations were determined as in [68]. The method is based on Waters AccQ.Tag Chemistry (Waters Corp., Milford. MA. Usa). The sample was lyophilized and finely ground in mortar. 10 mg of ground sample was extracted for 2 h with 1 ml of 20 mM HCl. Samples were then derivatized as such, diluted 1+1 with 20 mM HCl and diluted 1+1 with 0.032 mM amino acid standard solution in 20 mM HCl. The derivatization was carried out according to Waters AccQ.Tag manual.

The chromatography instrument consisted of Alliance 2690 Separation module and M-474 fluorescence detector monitoring at λ_ex. _= 248 nm/λ_em. _= 395 nm. The system was controlled and data treated with Empower chromatography software. All the instruments and derivatization reagent were from Waters corp. (Milford, MA. USA). For separations Carbamate analysis column was used. The eluent consisted of 140 mM sodium acetate +17 mM trietylamine pH 4.95 (solvent A) and 60% (w/w) acetonitrile:water (solvent B). The flow-rate was 1.5 ml/min and the column temperature was 37°C. The gradient was 0–2% B in 0.5 min; 2–7% B in 16.6 minutes; 7–13% B in 6 minutes; 13–34% B in 14 minutes, 34–34% B for 5 minutes with final washing step of 100% B for 2 minutes. All gradients were linear. Injection volume was 10 μL.

## Authors' contributions

MA carried out cDNA-subtraction library, cDNA-AFLP and Northern analysis and all data analysis and drafted the manuscript. TP participated in the design and coordination of the study and in drafting of the manuscript. KL carried out bioreactor cultivations and growth and protein production analysis from them and helped to draft the manuscript. MS participated in the design and coordination of the study and helped to draft the manuscript. MV participated in the design of the study and carried out cultivations and molecular genetics related to the ire1 overexpressing strain. TS carried our HPLC analysis of amino acids and helped to draft the manuscript. GR participated in conceiving of the study, in its design and in its coordination. MP conceived of the study, and participated in its design and coordination and helped to draft the manuscript.

## Supplementary Material

Additional File 1 A tab limited text file including: 1. List of experiment titles in *S. cerevisiae *gene expression plots. 2. Rules to divide ORFs having significant p-values into groups. 3. Systematic names of ORFs in the groups Click here for file

Additional File 2 Plot of the gene expression values of the three gene groups not discussed in the article. Click here for file

Additional File 3 A tab limited text file including: 1. Mapping of ESTs to *T. reesei *genome and homologues in *S. cerevisiae *or other species Click here for file

## Figures and Tables

**Table 1:** Representation of different function and location categories of genes in the different groups of secretion stress related genes of *S. cerevisiae*. Number of *S. cerevisiae *genes belonging to selected function and location categories within the different groups of secretion stress related genes distinguished in this article. The groups of differentially regulated genes: "coreUPR", "UPR Dependent Up "UPR Dependent Down", UPR Independent Up" and "UPR Independent Down", are defined in Materials and methods of this study and "UPR by Travers & al. 2000" (UPR regulated genes) are as described by [2], and "Homologues of *T. reesei *secretion stress ORFs" (numbers of ORFs corresponding to ESTs represented in the cDNA subtraction library and cDNA-AFLP analysis in this study). Category assignments are from CYGD functional or localization categories [51].

Topic	Category	cUPR	UPR Dependent UP	UPR Dependent DOWN	Not UPR Dependent UP	Not UPR Dependent DOWN	UPR by Travers & al. 2000	Homologues of T. reesei ER stress ORFs	*S. cerevisiae *genome
Protein secretion related	Cellular export and secretion			2	11	3	3	7	76
	Protein fate	23	30	25	137	65	95	62	1130
	Lipid, fattyacid and isoprenoid metabolism	6	10	5	33	18	28	16	271
	Vacuolar transport	2	2	4	26	9	10	11	152
	Vesicular transport	8	5	3	23	7	27	20	197
	Biogenesis of cellular componentsCell wall	2	8	4	21	11	21	16	212
	**% of Protein secretion related genes in the group**	**63%**	**31%**	**20%**	**34%**	**16%**	**38%**	**39%**	**26%**
Transcription related	Transcriptional control	1	23	8	44	33	26	23	491
	Transcription	3	35	19	69	140	48	31	1060
	**% of Transcription related genes in the group**	**7%**	**26%**	**13%**	**12%**	**23%**	**13%**	**14%**	**17%**
Outside of cell	Extracellular			1	2	9	5	5	50
	Cellwall				2	5	1	3	40
	**% of genes Outside of cell in the group**	**0%**	**0%**	**1%**	**1%**	**2%**	**1%**	**2%**	**1%**
Ribosome biogenesis			2	3		130	3	20	301
Histones								3	8
% of Unclassifed genes in the group		**20%**	**21%**	**40%**	**27%**	**17%**	**27%**	**9%**	**32%**
Total number of genes in the group		46	135	155	592	603	381	249	6335

**Table 2:** The most abundant ORFs in the secretion stress related ESTs of *T. reesei*. 25 ORFs represented by the highest number of ESTs in the cDNA subtraction libraries or cDNA-AFLP fragments derived from the secretion stress conditions are listed. Gene names from *T. reesei *(small case letters) or *S. cerevisiae *(capital letters) are shown. Description and *T. reesei *protein model identifiers (ID) are from *T. reesei *genome version 1.2 website. The number of ESTs corresponding to the genes in the libraries are shown: "AFLP", fragments from cDNA-AFLP analysis induced in both tPA expression and DTT treatment; "DTThyb1", subtraction library from DTT treated culture after one subtraction round; "DTTHyb5", subtraction library from DTT treated culture after five subtraction rounds; "tPAhyb1", subtraction library from the tPA producing culture after one subtraction round; "tPAHyb5", subtraction library from tPA producing culture after five subtraction rounds.

Gene	*T. reesei *protein Model ID	Description	AFLP	DTThyb1	DTThyb5	tPAhyb1	tPAhyb5	Total
TEF2	tre41793	translational elongation		25	12	42	18	97
bip1	tre42955	HSP70 family ER chaperone	1	55	1	1		58
	tre42349	posttranslational modification, protein turnover		40	15	1	2	58
UBI4	tre45056	posttranslational modification, protein turnover		46	2	1	2	51
cbhII	tre44954	cellobiohydrolase II				40	10	50
FRE4	tre39576	Ferric reductase-like transmembrane component		26		7		33
URA8	tre13936	phospholipid biosynthesis, pyrimidine base biosynthesis		1			32	33
cbhI	tre22421	cellobiohydrolase I				24	8	32
cpc1	tre37844	basic-leucine zipper (bZIP) transcription factor		21		10		31
	tre44646	unknown		13	12	1	1	27
	tre43768	ankyrin		13	11		2	26
	tre46310	putative exoglucanase		1		8	15	24
ACT1	tre41819	actin		8	2	8	5	23
ITR2	tre43977	intergal membrane myo-inositol transporter	1			16	2	19
	tre45769	aromatic compound metabolism		19				19
	tre43393	ADP-ribosylglycohydrolase		9	8		1	18
prp2	tre42899	putative PDI-related protein		2		3	13	18
MBF1	tre42770	transcription factor MBF1		13	3		1	17
HTA2	tre35558	histone H2A		9	4	1	2	16
YLL023C	tre43299	putative ER membrane protein		10	5			15
pdi1	tre45146	ER-resident foldase		12		2		14
cip1	tre41957	esterase homologue				6	8	14
	tre45203	Transcriptional coactivator p100		4	7	2	1	14
hex1	tre45186	woronin body		1		10	2	13
VPS1	tre44901	vacuolar sorting protein		12			1	13
Amount of ESTs sequenced from the individual libraries			94	961	240	510	242	2047
