# Supplementary figures and images for: Common features and interesting differences in transcriptional responses to secretion stress in the fungi Trichoderma reesei and Saccharomyces cerevisiae
Source: BMC Genomics. 2006 Feb 22;7:32. doi: 10.1186/1471-2164-7-32 (PMC1397821; doi:10.1186/1471-2164-7-32)

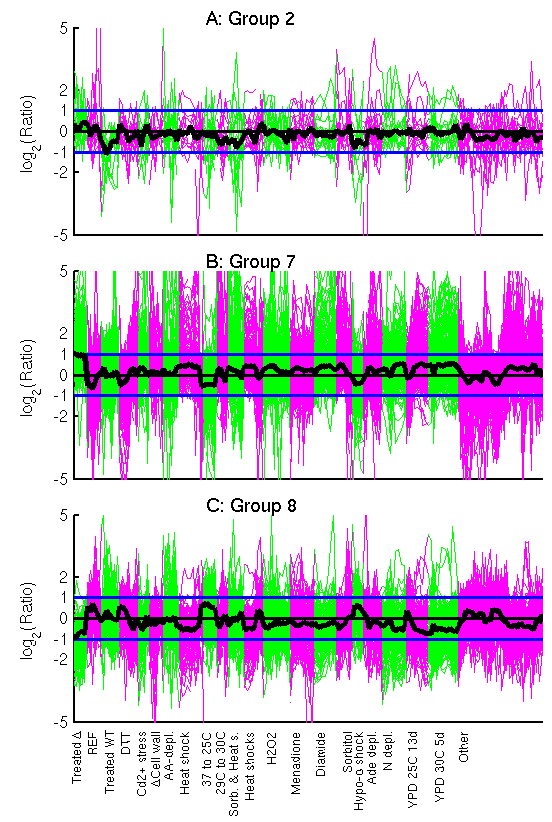

Supplement: Additional File 2 — Plot of the gene expression values of the three gene groups not discussed in the article. [file 1471-2164-7-32-S2.png]
